# Mimicking Cdk2 phosphorylation of Bcl-xL at Ser73 results in caspase activation and Bcl-xL cleavage

**DOI:** 10.1038/cddiscovery.2016.1

**Published:** 2016-02-01

**Authors:** NS Seng, J Megyesi, A Tarcsafalvi, PM Price

**Affiliations:** 1 Department of Physiology and Biophysics, University of Arkansas for Medical Sciences, Little Rock, AR, USA; 2 Division of Nephrology, Department of Internal Medicine, University of Arkansas for Medical Sciences, Little Rock, AR, USA; 3 Central Arkansas Veterans Healthcare System, Little Rock, AR, USA

## Abstract

Cisplatin is a widely used chemotherapeutic agent, yet its efficacy is limited by nephrotoxicity. The severity of nephrotoxicity is associated with the extent of kidney cell death. Previously, we found that cisplatin-induced kidney cell death was dependent on Cdk2 activation, and inhibition of Cdk2 protected cells from cisplatin-induced apoptosis. Using an *in vitro* kination assay, we showed that Cdk2 phosphorylated Bcl-xL, an anti-apoptotic member of Bcl-2 family proteins, at serine 73. We also found that this phosphorylated Bcl-xL participated in cell death, as a phosphomimetic mutant of Bcl-xL at the serine 73 site (S73D-Bcl-xL) activated caspases. We now find that S73D-Bcl-xL was cleaved at D61 and D76, which are putative caspase cleavage sites, to generate 15-kDa and 12-kDa fragments. Unlike full-length Bcl-xL, these cleavage products of Bcl-xL were previously reported to be pro-apoptotic. We sought to determine whether these Bcl-xL fragments were necessary for the induction of cell death by S73D-Bcl-xL. Mutation of these caspase cleavage sites prevented the formation of the 15-kDa and 12-kDa Bcl-xL cleavage products, but apoptosis still persisted in a S73D modified Bcl-xL. Our findings show that Cdk2 phosphorylation of Bcl-xL at Ser73, but not the Bcl-xL cleavage products, is necessary and sufficient to induce cell death.

## Introduction

Cis-diamminedichloroplatinum (II) or cisplatin has been widely used to treat various solid tumors, such as ovarian, testicular, head and neck, lung, and bladder cancers.^1^ In addition to causing cancer cell death, cisplatin also induces kidney cell death, and this nephrotoxicity is a major dose limiting factor of cisplatin in chemotherapy.^2^ Understanding the molecular mechanisms of cisplatin nephrotoxicity is important to find therapy to protect kidneys without compromising its anti-neoplastic effect.

Cyclin-dependent kinase 2 (Cdk2) is primarily known as a cell-cycle regulatory protein that participates in cell proliferation. Apart from its role in cell-cycle regulation, its role in cell death pathways is poorly understood but has become increasingly recognized.^3–7^ Our laboratory found that cisplatin-induced kidney cell death was dependent on Cdk2 activation, and inhibition of Cdk2 by p21, dominant-negative (DN)-Cdk2, or chemical inhibitors such as roscovitine or purvalanol protected kidney cells from cisplatin-induced apoptosis both *in vitro* and *in vivo*.^8–10^ However, the mechanisms of how Cdk2 mediates apoptosis are still unknown. Using an analog-sensitive Cdk2 kinase (F80G-Cdk2), we found that after cisplatin treatment, Cdk2 phosphorylated Bcl-xL, a cell survival protein, at a previously unreported Ser73 site.^11^

Although wild-type Bcl-xL (WT-Bcl-xL) inhibits pore formation induced by active Bax/Bak,^12–14^ a phosphomimetic Bcl-xL in which Ser73 was replaced by Asp promoted mitochondrial permeabilization, caspase activation, and apoptosis.^11^ WT-Bcl-xL was reported to be cleaved after Asp61 and Asp76 (D61 and D76) by caspases.^15,16^ The role of Bcl-xL cleavage in apoptosis is not well understood. It is unclear whether Bcl-xL cleavage results in loss of anti-apoptotic function,^16^ or whether the cleavage products could actively participate in apoptosis.^15,17,18^

In the present study, we report that Cdk2 phosphorylation of Bcl-xL at Ser73 is an upstream event resulting in perinuclear mitochondrial clustering, caspase activation, and subsequent Bcl-xL cleavage after D61 and D76. Furthermore, we show that Ser73 phosphorylation of Bcl-xL, but not Bcl-xL cleavage is necessary and sufficient to trigger apoptosis.

## Results

### Expression of phosphomimetic Bcl-xL at Ser73 resulted in 15 kDa and 12 kDa cleavage products from caspase activity

**Figure 1:**
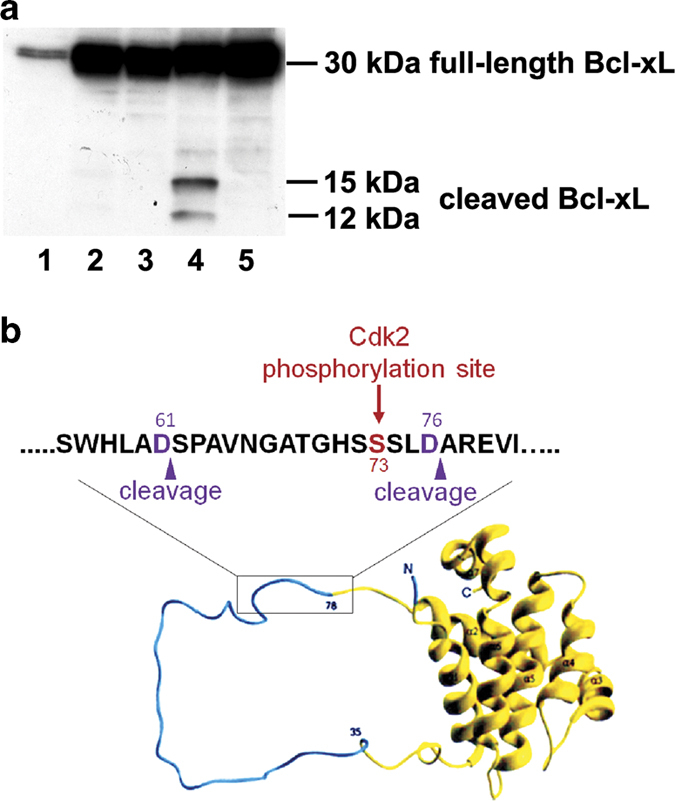
Phosphomimetic Bcl-xL (S73D-Bcl-xL) resulted in 15-kDa and 12-kDa cleavage products, which were eliminated by pan-caspase inhibitor, zVAD-fmk. (**a**) Cellular proteins were processed from untreated, untransduced TKPTS cells (lane 1), TKPTS cells transduced for 72 h with WT-Bcl-xL (lane 2), with WT-Bcl-xL in the presence of 5 *μ*M zVAD-fmk (lane 3), with S73D-Bcl-xL (lane 4), and with S73D-Bcl-xL in the presence of 5 *μ*M zVAD-fmk (lane 5). Western blot analysis was performed to analyze for full-length Bcl-xL (30 kDa) and Bcl-xL cleavage products (15 and 12 kDa). (**b**) Ribbon diagram of Bcl-xL showing the unstructured loop domain (cyan) and the boxed region within this domain indicate the approximate positions where Cdk2 phosphorylation of Bcl-xL (Ser73) and caspase cleavage of Bcl-xL (D61 and D76) occur.

Using western blot analysis, we determined the expression of Bcl-xL before and after transduction (Figure 1a). In comparison with the level of endogenous full-length Bcl-xL in untransduced cells (Figure 1a, lane 1), transduction resulted in a several fold increase in full-length Bcl-xL level (Figure 1a, lanes 2–5). In addition to full-length Bcl-xL, we also detected 15 kDa and 12 kDa Bcl-xL cleavage products in S73D-Bcl-xL-expressing cells (Figure 1a, lane 4), but not in WT-Bcl-xL-expressing cells (Figure 1a, lanes 2 and 3). Pretreatment of cells with 5 *μ*M pan-caspase inhibitor, zVAD-fmk, did not have any detectable effect on WT-Bcl-xL-expressing cells (Figure 1a, lane 3), but it eliminated the cleavage products of Bcl-xL from cells expressing S73D-Bcl-xL (Figure 1a, lane 5), suggesting the involvement of active caspase(s) in the formation of Bcl-xL cleavage products. The Cdk2 phosphorylation site (Ser73) and the reported caspase cleavage sites (D61 and D76) are indicated in the ribbon diagram of Bcl-xL, adapted from Muchmore *et al.* ^19^ (Figure 1b).

### Endogenous Bcl-xL was cleaved in response to cisplatin exposure

**Figure 2:**
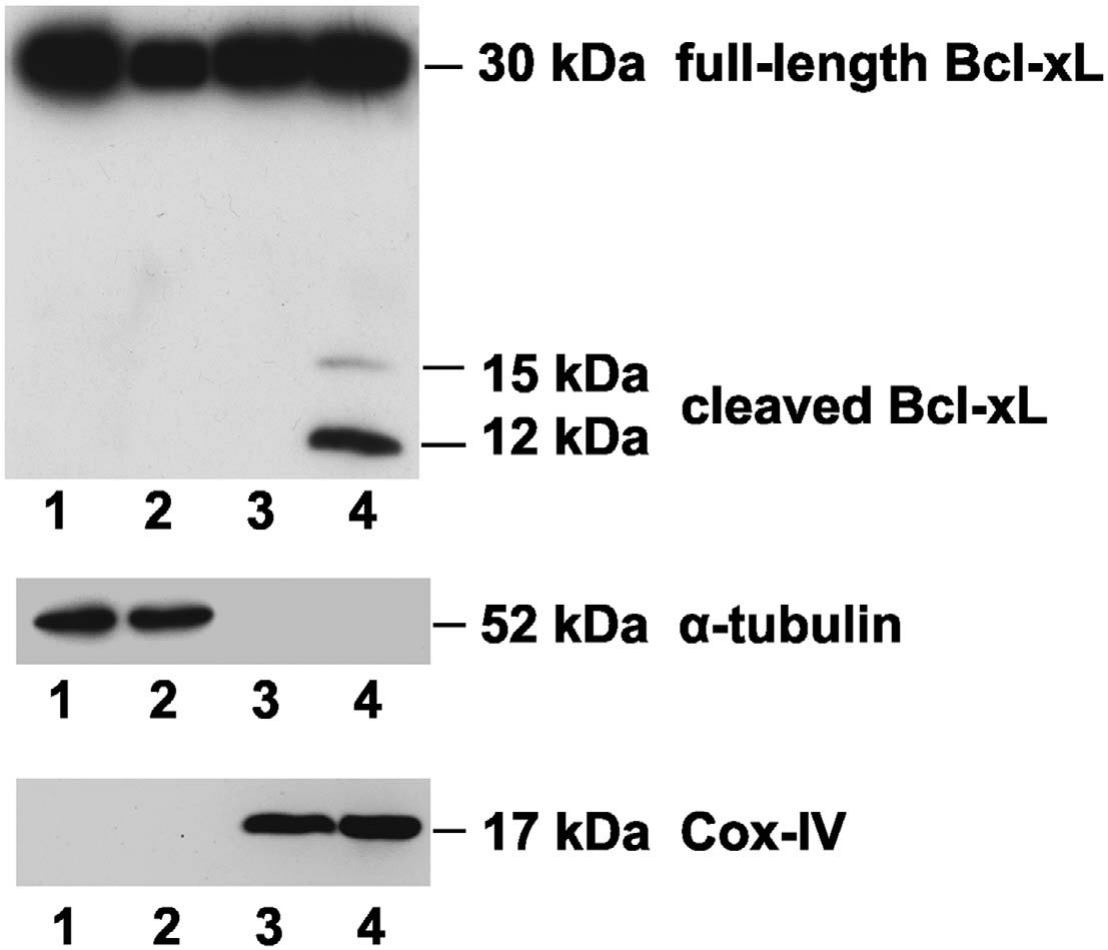
Cisplatin induced cleavage of Bcl-xL. TKPTS cells were either untreated (lanes 1 and 3) or treated with 25 *μ*M cisplatin for 24 h (lanes 2 and 4). Untreated control and cisplatin-treated TKPTS cells were processed into supernatant (lanes 1 and 2) and membrane fractions (lanes 3 and 4) as described in Materials and Methods. Western blot analysis was performed to detect full-length Bcl-xL (30 kDa) and cleaved Bcl-xL (15 and 12 kDa). Alpha-tubulin and cytochrome c oxidase subunit IV (COX-IV) were used as markers to show the purity of the fractionations of supernatant and membrane containing mitochondria.

As S73D-Bcl-xL mimics the phosphorylation of Bcl-xL by Cdk2 after cisplatin treatment, we investigated whether endogenous Bcl-xL would also be cleaved after cisplatin treatment. The cleavage of endogenous Bcl-xL was analyzed in the supernatant and membrane fractions of control untreated TKPTS cells and cisplatin-treated TKPTS cells (Figure 2). No cleavage products of Bcl-xL were observed in the control and cisplatin-treated supernatant fractions (Figure 2, lanes 1 and 2), but both 15-kDa and 12-kDa cleavage products were localized in the membrane fraction of cisplatin-treated TKPTS cells (Figure 2, lane 4). The cleavage products of Bcl-xL were absent in the membrane fraction of the control untreated cells (Figure 2, lane 3).

### Cleavage of endogenous Bcl-xL is dependent on active caspases downstream of Cdk2 activation

**Figure 3:**
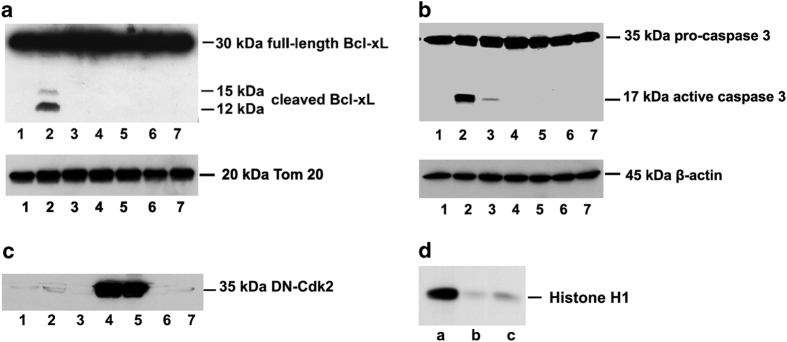
Endogenous Bcl-xL cleavage was associated with caspase 3 activation, and was downstream of Cdk2 activation. TKPTS cells were untreated and untransduced (lane 1) and treated with 25 *μ*M cisplatin for 24 h (lanes 2, 3, 5, and 7). To inhibit Cdk2 activity, TKPTS cells were transduced with 100 MOI DN-Cdk2 24 h before cisplatin exposure (lanes 4 and 5) or treated with 9 *μ*M purvalanol 1 h before cisplatin exposure (lanes 6 and 7). To inhibit caspase activity, 5 *μ*M zVAD fmk was added 1 h before cisplatin exposure (lane 3). After 24 h of cisplatin treatment, cells were processed into (**a**) mitochondria/membrane fraction for western blot analysis of Bcl-xL and Tom20 (loading control), (**b**) supernatant fraction for western blot analysis of caspase 3 and *β*-actin (loading control) and (**c**) DN-Cdk2. (**d**) Histone kinase assay was performed on untransduced TKPTS cells (lane a), TKPTS cells treated with purvalanol (lane b), and TKPTS cells transduced with DN-Cdk2 (lane c).

As the S73D-Bcl-xL cleavage products were eliminated by the pan-caspase inhibitor, zVAD-fmk (Figure 1a, lane 5), we hypothesized that the cleavage of endogenous Bcl-xL observed after cisplatin exposure would similarly be prevented by zVAD-fmk. The cleavage of Bcl-xL was not observed in the membrane fraction of the untreated control cells (Figure 3a, lane 1), but it was detected in that of cisplatin-treated TKPTS cells (Figure 3a, lane 2). The cleavage products of Bcl-xL generated in response to cisplatin treatment were prevented by pretreating cells with pan-caspase inhibitor, zVAD-fmk (Figure 3a, lane 3). In addition, the cleavage products were also eliminated by inhibition of Cdk2 either by DN-Cdk2 transduction (Figure 3a, lane 5) or by pretreatment of cells with purvalanol (Figure 3a, lane 7). In the absence of cisplatin, TKPTS cells either transduced with DN-Cdk2 (Figure 3a, lane 4) or pretreated with purvalanol (Figure 3a, lane 6) had no effect on the cleavage of endogenous Bcl-xL. Caspase 3 was activated in TKPTS cells in the presence of cisplatin (Figure 3b, lane 2). Pretreatment of cells with either zVAD-fmk (Figure 3b, lane 3) or inhibition of Cdk2 either by overexpression of DN-Cdk2 (Figure 3b, lane 5) or by pretreatment of cells with purvalanol (Figure 3b, lane 7) prevented cisplatin-induced caspase 3 activation. DN-Cdk2 overexpression was observed where TKTPS cells were transduced with DN-Cdk2 adenovirus (Figure 3c, lanes 4 and 5). Compared with control untransduced cells (Figure 3d, lane a), TKPTS cells transduced with DN-Cdk2 or pretreated with purvalanol (Figure 3d, lanes b and c, respectively) resulted in the inhibition of endogenous Cdk2 activity, as indicated by a reduction in the phosphorylation of histone H1, a known Cdk2 substrate.

### Cleavage-resistant phosphomimetic Bcl-xL at Ser73 preserved its ability to induce apoptosis

**Figure 4:**
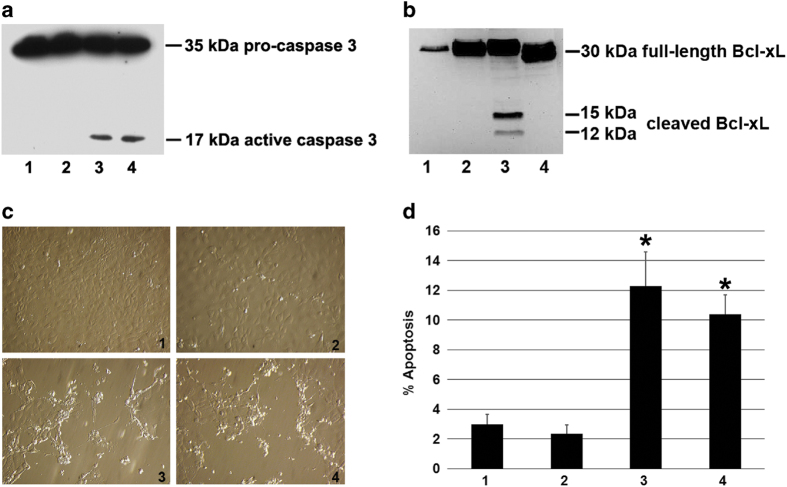
Caspase-resistant Bcl-xL (ADA-Bcl-xL) preserved its ability to induce apoptosis despite the lack of Bcl-xL cleavage products. TKPTS cells were untransduced (1), transduced for 72 h with WT-Bcl-xL (2), with S73D-Bcl-xL (3) and with ADA-Bcl-xL (4). Western blot analysis was performed to detect (**a**) pro-caspase 3 and active caspase 3 and (**b**) Bcl-xL and its cleavage products. (**c**) Cellular morphology of TKPTS cells was documented with Hoffmann Modulation Contrast microscope, ×20 objective. (**d**) The percentage of apoptosis (Sub G0/G1 region of cell-cycle analysis) was also quantified by using propidium iodide and flow cytometry. Data are represented by the mean±S.E.M. of at least three independent experiments. Statistical significance was calculated using two-tailed Student's *t*-test, with asterisk (*) representing *P*<0.05.

WT-Bcl-xL was reported to be cleaved by caspases after D61 and D76.^15,16^ To test whether S73D-Bcl-xL was cleaved at these residues, site-directed mutagenesis was performed to convert aspartic acids at 61 and 76 into alanines (that is, D61A and D76A), resulting in a triple mutated protein (D61**A**, S73**D**, and D76**A**) referred to hereafter as ADA-Bcl-xL. Caspase 3 activation was detected in S73D-Bcl-xL- and ADA-Bcl-xL-transduced cells (Figure 4a, lanes 3 and 4, respectively), but not in the untransduced control cells and cells transduced with WT-Bcl-xL (Figure 4a, lanes 1 and 2, respectively). Caspase activation is the cause of Bcl-xL cleavage (Figure 1a, lane 5 and 3a, lane 3).^15,16,20^ Bcl-xL cleavage was not detected in control untransduced cells and cells transduced with WT-Bcl-xL (Figure 4b, lanes 1 and 2, respectively) since caspase was not activated under those conditions (Figure 4a, lanes 1 and 2). The caspase activation resulted in 15-kDa and 12-kDa cleavage products in S73D-Bcl-xL transduced cells (Figure 4b, lane 3). Although caspase activity was detected in ADA-Bcl-xL-expressing cells (Figure 4a, lane 4), Bcl-xL cleavage products were not generated (Figure 4b, lane 4), suggesting that the cleavage sites were conserved at D61 and D76 in S73D-Bcl-xL, and substituting alanine residues on those sites abolished Bcl-xL cleavage. Control untransduced cells and WT-Bcl-xL transduced cells were morphologically similar, as represented by solid monolayers of healthy cells (Figure 4c, 1 and 2, respectively). Similar to S73D-Bcl-xL, ADA-Bcl-xL was able to induce apoptosis, which was evident by morphological changes such as cell shrinkage, membrane blebbing, and cell detachment (Figure 4c, 3 and 4, respectively). The percent of apoptosis in cells expressing ADA-Bcl-xL (10.40±0.61%, Figure 4d, bar 4) was significantly higher than the percent of apoptosis in control untransduced cells (2.98±0.66%, Figure 4d, bar 1) and cells expressing WT-Bcl-xL (2.35±0.60%, Figure 4d, bar 2), but not significantly different from the percent of apoptosis in cells expressing S73D-Bcl-xL (12.27±2.32%, Figure 4d, bar 3).

### ADA-Bcl-xL and S73D-Bcl-xL induce perinuclear clustering of mitochondria

**Figure 5:**
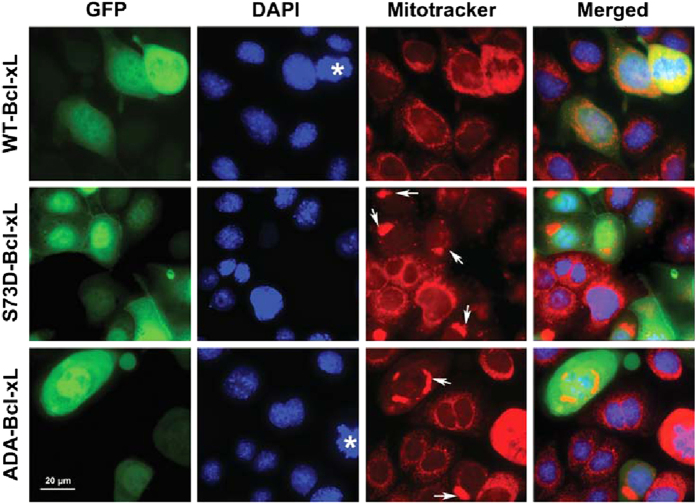
Expression of ADA-Bcl-xL and S73D-Bcl-xL resulted in perinuclear mitochondrial clustering. TKPTS cells transduced with WT-Bcl-xL, S73D-Bcl-xL, and ADA-Bcl-xL for 48 h were photographed using a Nikon Eclipse Ti-S fluorescent microscope. GFP expressing cells identified cells transduced with Bcl-xL. DAPI and Mitotracker Red CMXRos were used to stain nuclei and mitochondria, respectively. Asterisks represent dividing cells, which alters the intensity of GFP. White arrows indicate mitochondrial clustering. Merged pictures showed the perinuclear localization of clustered mitochondria in S73D-Bcl-xL- and ADA-Bcl-xL-expressing cells.

Mitochondrial morphology and subcellular distribution change during apoptosis; mitochondria are dispersed throughout the cytoplasm in normal cells, but are condensed and clustered in perinuclear regions in apoptotic cells.^21^ Using fluorescent microscopy, we determined the subcellular distribution of mitochondria in cells expressing WT-Bcl-xL, S73D-Bcl-xL, and ADA-Bcl-xL, using 4’,6-diamidino-2-phenylindole (DAPI) and Mitotracker Red CMXRos to mark nuclei and mitochondria, respectively. Cells expressing transduced Bcl-xL were identified by GFP fluorescence, as GFP was co-expressed in the adenoviral expression vectors. Mitochondria were dispersed throughout the cytoplasm in WT-Bcl-xL-expressing cells similar to untransduced cells (cells without GFP marker), but were clustered in the perinuclear region in S73D-Bcl-xL- and ADA-Bcl-xL-expressing cells (Figure 5), consistent with their localization in the early stages of apoptosis.

## Discussion

Bcl-xL contains an unstructured loop domain (amino acids, 26–83), which is a regulatory region of Bcl-xL,^22^ known to be modulated by phosphorylation^23,24^ and cleavage.^15,16^ Recently, we reported that in normal kidney cells, Bcl-xL was phosphorylated at Ser73 within the unstructured loop domain in response to cisplatin exposure.^11^ Ser73 phosphorylation converted Bcl-xL into a potent pro-apoptotic pore forming molecule, which oligomerized at the mitochondria to release cytochrome c and activate caspase 3.^11^ In addition, Ser73 phosphorylation promoted the cleavage of Bcl-xL, as evident by the formation of the 15-kDa and 12-kDa fragments specifically in cells expressing phosphomimetic S73D-Bcl-xL (Figure 1a, lane 4 and Figure 4b, lane 3), but not the wild-type Bcl-xL (Figure 1a, lane 2 and Figure 4b, lane 2). These cleavage products were resulted from upstream caspase activation as pretreatment of cells with zVAD-fmk prevented Bcl-xL cleavage (Figure 1a, lane 5).

In response to cisplatin exposure, endogenous Bcl-xL was cleaved into fragments similar to S73D-Bcl-xL fragments, suggesting that Bcl-xL cleavage is a relevant biological process in cisplatin-induced apoptosis. However, unlike the S73D-Bcl-xL-expressing cells (Figure 1a, lane 4 and Figure 4b, lane 3), cells exposed to cisplatin generated the 12-kDa cleavage product in higher quantity than the 15-kDa cleavage product (Figure 2, lane 4 and Figure 3a, lane 2). Such differences could be explained by the fact that S73D-Bcl-xL induced cell death represents only a subset of the many cell death pathways induced by cisplatin. It is possible that proteases that process Bcl-xL into the 12-kDa cleavage product were more activated in cisplatin-treated cells than in S73D-Bcl-xL-expressing cells.

As pan-caspase inhibitor, zVAD-fmk, prevented the endogenous cleavage of Bcl-xL (Figure 3a, lane 3), the cleavage of Bcl-xL is downstream of caspase activation. Cdk2 activation is upstream of caspase activation because inhibition of Cdk2 either by DN-Cdk2 (Figures 3a and b, lane 5) or by purvalanol (Figures 3a and b, lane 7) prevented caspase activation (caspase 3) and subsequent Bcl-xL cleavage. Expression of phosphomimetic S73D-Bcl-xL resulted in caspase activation,^11^ suggesting that Cdk2 phosphorylation of Bcl-xL at Ser73 is upstream of caspase activation, but downstream of Cdk2 activation.

The 15-kDa cleavage product of Bcl-xL has a similar molecular weight as Bcl-xS, a proapoptotic isoform of Bcl-xL, and we investigated whether the observed cleavage product could be Bcl-xS. Bcl-xS is produced from Bcl-x gene with BH1 and BH2 domains truncated due to alternative splicing of Bcl-x mRNA.^25^ On the other hand, Bcl-xL cleavage products are produced post-translationally by activated caspases, resulting in the loss of the BH4 domain.^15,16^ Since the formation of both the 15-kDa and 12-kDa Bcl-xL fragments could be prevented by zVAD-fmk, we concluded that those fragments were Bcl-xL cleavage products rather than Bcl-xS.

Bcl-xL was previously reported to be cleaved after D61 and D76 by caspases.^15,16^ Mutating these cleavage sites on S73D-Bcl-xL, that is, D61**A**, S73**D**, D76**A**-Bcl-xL (ADA-Bcl-xL) eliminated the 15-kDa and 12-kDa fragments (Figure 4b, lane 4). Despite the absence of Bcl-xL cleavage products, ADA-Bcl-xL was still able to induce caspase 3 activation (Figure 4a, lane 4) and apoptosis (Figure 4c, panel 4 and Figure 4d, bar 4), indicating that phosphorylation of Bcl-xL at Ser73 rather than its cleavage was necessary and sufficient to induce apoptosis.

Mitochondria are normally distributed throughout the entire cytoplasm.^21^ Perinuclear mitochondrial clustering has been reported in apoptotic cells induced by various stimuli such as TRAIL,^26^ exogenous expression of t-Bid^27^ and Bax,^21^ aluminum maltolate,^28^ and staurosporin.^28^ Although perinuclear mitochondrial clustering is a phenotype consistently associated with apoptosis, it is still not clear whether mitochondrial clustering facilitates cell death. It is speculated that condensation of mitochondria could mediate the release of apoptogenic factors from mitochondria, and perinuclear localization hastens the delivery of pro-apoptotic molecules from the mitochondria into the nucleus, thereby increasing the rate of apoptosis induction.^21^ Here, we show that ADA-Bcl-xL induces perinuclear clustering of mitochondria, similar to S73D-Bcl-xL (Figure 5). This suggests that Cdk2 phosphorylation of Bcl-xL was sufficient to cause mitochondrial redistribution and caspase activation, independent of Bcl-xL cleavage.

**Figure 6:**
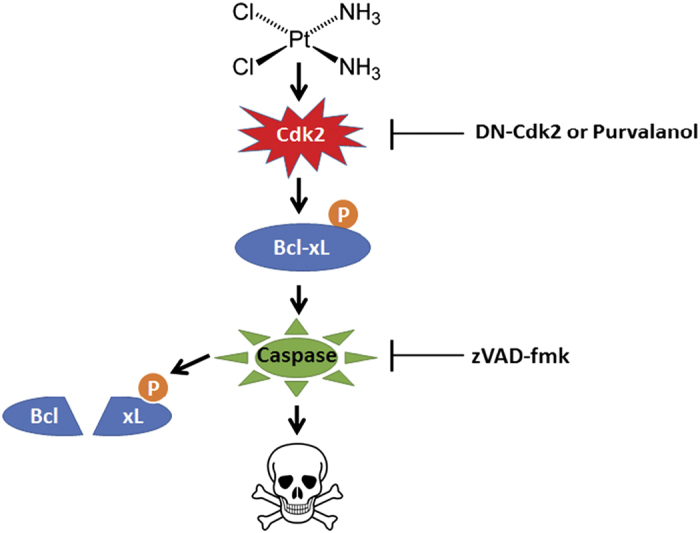
The proposed model of Cdk2 phosphorylation of Bcl-xL in cisplatin-induced apoptosis. Cdk2 activated in response to cisplatin treatment phosphorylated Bcl-xL at Ser73. Bcl-xL, when phosphorylated at Ser73, promoted self-oligomerization to engage in caspase-dependent mitochondrial pathway of apoptosis.^11^ In the process, active capases cleaved Bcl-xL at previously reported sites, D61 and D76. However, the resulting cleavage products of Bcl-xL were not necessary for apoptosis. Phosphorylation of Bcl-xL at Ser73 was sufficient to induce cell death. Inhibition of Cdk2 using DN-Cdk2 or purvalanol and inhibition of active caspases using zVAD-fmk blocked caspase activation, and subsequent Bcl-xL cleavage.

Our data suggest that Cdk2 phosphorylation of Bcl-xL at Ser73 is an important part of cisplatin-induced kidney cell death pathways (Figure 6). Caspases, activated by phosphomimetic S73D-Bcl-xL, proteolyzed Bcl-xL into the 15-kDa and 12-kDa cleavage products. Elimination of the caspase cleavage sites resulted in the absence of the Bcl-xL cleavage products, but did not prevent mitochondrial redistribution, caspase activation, and subsequent apoptosis, indicating that the cleavage products were not essential for apoptosis induction. However, the differences in the intensities of the Bcl-xL cleavage products between cisplatin-induced apoptosis and S73D-Bcl-xL-induced apoptosis suggest that the mechanisms of how the cleavage products are generated could be regulated by more than one pathway. It is known that cisplatin triggers multiple cell death pathways,^2,29^ and Cdk2 phosphorylation of Bcl-xL represents one of these modes. Further investigations on how the cleavage products are generated, especially in cisplatin-treated cells, could offer insights into mechanisms of cisplatin-induced cell death.

## Materials and methods

### Cell culture

Mouse kidney proximal tubule cells (TKPTS)^30^ were grown in DMEM+Ham’s F-12 culture medium supplemented with 50 μU/ml insulin and 7% FBS at 37 °C in 5% CO_2_. At 75% confluency of cells, cisplatin (APP Pharmaceuticals, Schaumburg, IL, USA) was added, where indicated, to a final concentration of 25 *μ*M. Purvalanol A (540500, Calbiochem, San Diego, CA, USA) and zVAD-fmk (627610, Calbiochem, San Diego, CA, USA) were added 1 h before cisplatin treatment to 9 and 5 *μ*M, respectively, where indicated. Adenovirus expressing wild-type Bcl-xL, phosphomimetic Bcl-xL (S73D-Bcl-xL), and cleavage-resistant phosphomimetic Bcl-xL (D61**A**, S73**D**, D76**A**-Bcl-xL) were added, where indicated, at a final multiplicity of infection (MOI) of 100. For adenoviral transduced cells, zVAD-fmk was added to the cultures to 5 *μ*M at day 0, day 1, and day 2, where indicated. Cells were collected for analyses on day 3 after viral transduction.

### Cell-cycle analysis

TKPTS cells were trypsinized and collected by centrifugation at 800×*g* for 7 min. The cells were processed for propidium iodide (PI) staining as previously described.^8^ Cell-cycle analysis was performed using FACSCalibur for 10 000 cells per experiment, and analyzed by FlowJo (Ashland, OR, USA). Percent apoptosis was quantified by the cell population in sub G0/G1 region^31^ using at least three independent experiments.

### Western blot

Cells were lysed in RIPA lysis buffer (150 mM NaCl, 50 mM Tris HCl pH 7.5, 0.1% SDS, 1% NP-40, 0.5% sodium deoxycholate, and 1 mM EDTA) supplemented with protease inhibitor cocktail (P8340, Sigma-Aldrich, St Louis, MO, USA). Lysates were sonicated, and centrifuged at 13 000 r.p.m. for 10 min to remove cell debris. Protein concentrations were determined by Bradford assay using Bio-Rad Protein Assay Dye Reagent Concentrate (500-0006, Bio-Rad, Hercules, CA, USA). Equal amount of samples was separated on SDS-PAGE, transferred onto 0.2 *μ*m nitrocellulose membrane (162-0097, Bio-Rad), and probed with primary antibody against the protein of interest overnight at 4 ºC. Then, the membrane was incubated with appropriate HRP-conjugated secondary antibody. The presence of the protein of interest was detected by Clarity Western ECL Substrate (170-5061, Bio-Rad), and visualized by enhanced chemiluminescence film (28906837, GE Healthcare, Pittsburgh, PA, USA). Primary antibodies used in this study were human/mouse Bcl-x polyclonal antibody (AF800, R&D Systems, Minneapolis, MN, USA), caspase 3 (9662, Cell Signaling, Danvers, MA, USA), cleaved caspase 3 (9661, Cell Signaling), Cdk2 (ab7954, Abcam, Cambridge, MA, USA), α-tubulin (3873, Cell Signaling), *β*-actin (4970, Cell Signaling), Tom20 (sc-11415, Santa Cruz Biotechnology, Santa Cruz, CA, USA), and cytochrome c oxidase subunit IV (4844, Cell Signaling). HRP-linked anti-rabbit IgG (NA934V, GE Healthcare), and HRP-linked anti-mouse IgG (NXA931, GE Healthcare) were used as the secondary antibodies in this study.

### Histone kinase assay

Untransduced TKPTS cells and TKTPS cells either transduced with 100 MOI DN-Cdk2 adenovirus for 24 h or treated with 9 *μ*M purvalanol for 1 h were lysed in NP-40 lysis buffer (150 mM NaCl, 50 mM Tris pH 7.5, 1% NP-40 and 0.5 mM EDTA) supplemented with protease inhibitor cocktail. *In vitro* kinase assay was performed using histone H1 (Upstate Biotechnology, Billerica, MA, USA) as a Cdk2 substrate as previously described.^32^

### Subcellular fractionation

Cells were harvested using CHAPS lysis buffer (50 mM HEPES, pH 7.4, 5 mM CHAPS, and 5 mM DTT) supplemented with protease inhibitor cocktail. Cell lysate was homogenized by douncing and centrifuged at 16 000×*g* to separate membranes from supernatant. To analyze membrane-associated proteins, the membrane fraction was further sonicated in RIPA lysis buffer, supplemented with protease inhibitor cocktail.

### Site-directed mutagenesis

Site-directed mutagenesis was performed by using the Q5-Site-directed mutagenesis kit (E0554S, New England Biolabs, Ipswich, MA, USA) according to the manufacturer’s protocol. D61A mutagenesis was generated by using the forward and reverse primers 5′GCACCTGGCGGCTAGCCCGGCCG3’ and 5′CAGGATGGGTTGCCATTGATGGCACTGG3′, respectively. D76A mutation was generated by the forward and reverse primers, 5′CAGCAGTTTGGCTGCGCGGGAGG3′ and 5′CTGTGGCCAGTGGCTCCATTC3′, respectively. To create D61A, D76A double mutation, D61A mutation was created after D76A mutation was performed in S73D-Bcl-xL plasmid. All mutations were confirmed by DNA sequencing, performed at the University of Arkansas for Medical Sciences core facility.

### Fluorescent microscopy

TKPTS cells transduced for 48 h with WT-Bcl-xL, S73D-Bcl-xL, and ADA-Bcl-xL were labeled with 100 nM Mitotracker Red CMXRos (Molecular Probes, Eugene, OR, USA) for 1 h at 37 ºC, fixed, and then processed as previously described.^11^ DAPI-containing mounting media (Vector, Burlingame, CA, USA, H1500) was used to label nuclei. Images were acquired using Nikon Eclipse Ti-S (Nikon, Melville, NY, USA), apochromatic ×60 oil lens and NIS Elements imaging software (Nikon).

### Adenovirus

Adenoviruses for WT-Bcl-xL, S73D-Bcl-xL, and DN-Cdk2 used in this study were generated as described.^10,11,33^ Using a similar strategy, ADA-Bcl-xL adenovirus was generated for this study. In brief, site-directed mutagenesis was performed to generate D61A and D76A in the S73D-Bcl-xL plasmid as described above. The cDNA fragment containing the triple mutation (D61A, S73D, and D76A) was excised from the plasmid using restriction enzymes *Hin*dIII and *Eco*RI to replace a similar fragment in pAd-Track-CMV-WT-Bcl-xL. ADA-Bcl-xL adenovirus was generated in AD-293 cells, amplified in HEK-293 cells, and purified using CsCl banding as described.^34^

### Statistical analysis

All quantitative measures were expressed in mean±S.E.M. Probabilities of statistical significance were determined using the two-tailed *t*-test for independent variables with data from at least three separate experiments. Statistical significance was defined as *P*<0.05.

## Abbreviations

Cdk2: Cyclin-dependent kinase 2
DAPI: 4′,6-diamidino-2-phenylindole
DN: dominant-negative
TKPTS: mouse kidney proximal tubule epithelial cells.
